# Structural and Evolutionary Analysis of *Saci2*-Like LTR Retrotransposons in Diphyllobothriidean Tapeworms

**DOI:** 10.3390/ijms26189061

**Published:** 2025-09-17

**Authors:** Young-An Bae

**Affiliations:** Department of Microbiology and Lee Gil Ya Cancer and Diabetes Institute, Gachon University College of Medicine, Incheon 21999, Republic of Korea; yabae03@gmail.com

**Keywords:** LTR retrotransposon, *Saci2*-like retrotransposon, retrotransposon dynamics, genome evolution, *Spirometra erinaceieuropaei*, *Sparganum proliferum*, tapeworms

## Abstract

Cyclophyllideans, which diverged from diphyllobothriideans, have evolved compact genomes to meet ecological and biological demands associated with rapid development, early maturation, and prolific asexual reproduction. This streamlining is accompanied by inactivation of transposable elements (TEs), including retrotransposons. In contrast, diphyllobothriideans retain large, retrotransposon-rich genomes, but information on their individual retrotransposons is lacking. Here, *Saci2*-like long terminal repeat (LTR) retrotransposons, formerly annotated as *lennie* in taeniid cestodes, were identified in the diphyllobothriideans *Spirometra erinaceieuropaei* and *Sparganum proliferum*, along with orthologs from *Schistocephalus solidus* and *Ligula intestinalis*. The *Saci2* homologs in these genomes diversified into at least eight families, exhibiting substantial variation in LTR and primer binding site sequences, reflecting ongoing regulatory diversification. Phylogenetic and divergence analyses indicated that they maintain structural and functional integrity under purifying selection, while early signs of inactivation appeared in *S. proliferum*. These findings suggest that diphyllobothriideans have faced little pressure for genome compaction, permitting the retention of functional retrotransposons, whereas cyclophyllideans, particularly taeniids, underwent genome streamlining linked to shortened life cycles and high fecundity, resulting in retrotransposon degradation. This contrast underscores the reciprocal relationship between biological demands and genome remodeling with TE inactivation in metazoans.

## 1. Introduction

Cestodes, or tapeworms, are digenean endoparasites that require at least two different host species to complete their life cycle. After hatching, the embryos of these parasites, known as oncospheres, develop into metacestodes in invertebrate or vertebrate intermediate hosts. They subsequently mature into sexually active adults in other vertebrate definitive hosts. As members of the phylum Platyhelminthes and class Cestoda, tapeworms are subdivided into two subclasses: monozoic (unsegmented) Cestodaria and polyzoic (segmented) Eucestoda. The latter encompasses the vast majority of known tapeworm species and categorized into several orders such as Cyclophyllidea and Diphyllobothriidea, on the basis of morphology, developmental characteristics, and life-cycle patterns [1,2].

The orders Cyclophyllidea and Diphyllobothriidea contain species of medical importance. Cyclophyllideans primarily infect humans through the ingestion of infected meat or other intermediate hosts, while diphyllobothriideans are acquired by consuming raw or undercooked fish, reptiles, or amphibians [2,3]. These two groups can be distinguished from each other by a range of characteristic traits. These include scolex type (suckers vs. bothria), egg type (hexacanth embryo with hooklets vs. operculated egg), egg-release strategy (shedding of entire gravid proglottids vs. gradual release through a uterine pore), and typical host range (terrestrial vertebrates vs. aquatic vertebrates and some mammals). One of the most striking biological differences lies in the capacity for asexual reproduction [2]. Many cyclophyllideans, particularly members of the family Taeniidae, asexually produce thousands of protoscoleces, which develop into new individuals, during their larval stages through processes such as budding or fragmentation [4]. In contrast, diphyllobothriidean larvae generally lack this proliferative capability, with the rare exception of *Sparganum proliferum*. The larvae of *S. proliferum* form medusa-like branching plerocercoids capable of somatic proliferation in human tissues [5,6]. Taken together, the bulk dissemination of immediately infective eggs and the intense asexual proliferation observed in cyclophyllideans may impose considerable demands on ecological adaptation and biosynthetic capacity. These demands are particularly strong in relation to their high fecundity and rapid maturation. Given that Diphyllobothriidea represents a basal or more ancestral lineage relative to Cyclophyllidea [1], it is plausible that cyclophyllideans have evolved additional strategies, potentially including genomic streamlining, to meet these heightened biological challenges.

Recent advances in genome sequencing have enabled detailed comparisons between the major cestode lineages, diphyllobothriideans such as *Spirometra erinaceieuropaei* (Rudolphi, 1819) Mueller, 1937, *S. proliferum*, and *Schistocephalus solidus*, and cyclophyllideans including *Echinococcus granulosus*, *Taenia solium*, and *Hymenolepis microstoma* [6,7]. These genomic datasets reveal striking contrasts in size, structure, and repetitive content, reflecting their divergent evolutionary strategies. Diphyllobothriidean genomes are substantially larger (539.4 Mb in *S. solidus* to 796 Mb in *S. erinaceieuropaei*, Japanese isolate) than those of cyclophyllideans (114.9 Mb in *E. granulosus* to 141.1 Mb in *H. microstoma*), a difference partly attributable to the reduction in both the number and average length of protein-coding genes in cyclophyllidean genomes [6]. However, the primary factor influencing genome size variation is likely the differential abundance of repetitive sequences, including transposable elements (TE). In diphyllobothriideans, repeats constitute approximately 50% of the genomes, whereas they account for only 7–10% in cyclophyllideans. Among these repeats, non-long terminal repeat (non-LTR; ≈ 31.5%) and LTR retrotransposons (≈5%) are the most and second-most abundant classes, respectively, in diphyllobothriidean genomes. In contrast, cyclophyllidean genomes are dominated by LTR retrotransposons (0.7–1.3%) and DNA transposons (0.3–1.1%), aside from non-typeable repeats [6,7]. These patterns suggest that TEs in cyclophyllideans have undergone extensive inactivation and subsequent decay in parallel with genome compaction, after diverging from diphyllobothriideans. The pronounced reduction in genome size may be a consequence of strong selective pressures favoring genomic streamlining, potentially driven by developmental, metabolic, and ecological constraints, including those associated with high-burden larval proliferation.

Retrotransposons are broadly classified into non-LTR and LTR types, which differ in both structure and integration mechanisms [8,9]. Of these, LTR retrotransposons possess terminal repeats containing potent promoter, enhancer, and polyadenylation signals, enabling them to influence host gene expression even after partial decay into solo LTRs [10,11]. These residual LTRs can serve as alternative promoters or enhancers for nearby genes. They can also act as hotspots for homologous recombination, leading to chromosomal rearrangements and large-scale deletions that contribute to genome compaction [12,13]. Such effects extend beyond simple genome expansion, positioning LTR retrotransposons as major drivers of genome remodeling [14,15].

The taxonomic status of *Sparganum proliferum* remains unresolved. It is generally regarded as an aberrant larval form related to the genus *Spirometra*, lacking a confirmed adult stage and showing atypical growth through asexual proliferation in humans and other hosts [6]. While its precise classification is uncertain, comparative genomic data nonetheless support its taxonomical position as the closest relative of *S. erinaceieuropaei*. Most LTR retrotransposons identified in cestode genomes belong to the *Ty3/gypsy* superfamily [6,7,16]. To date, only a single LTR retrotransposon, *lennie*, has been characterized in cyclophyllidean cestodes [16,17], whereas individual retrotransposons remain undescribed in diphyllobothriidean species. In the present study, *lennie*-like LTR retrotransposons were isolated from the non-proliferative *S. erinaceieuropaei* and the proliferative *S. proliferum*. In addition to structural characterization, their evolutionary dynamics were examined in combination with their orthologs from other diphyllobothriideans, including *S. solidus* and *L. intestinalis*, to assess their contribution to the contrasting trajectories of genome evolution in diphyllobothriidean and cyclophyllidean lineages.

## 2. Results

### 2.1. Retrievals of Eg_lennie Pol Homologs in S. erinaceieuropaei

**Figure 1:**
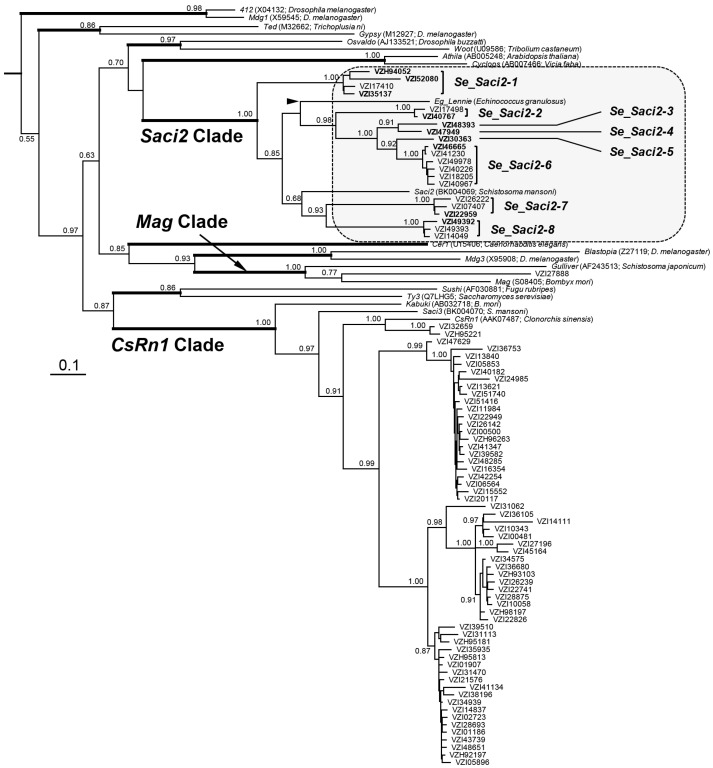
Phylogeny of long terminal repeat (LTR) retrotransposons identified in *Spirometra erinaceieuropaei*. Representative LTR retrotransposons from distinct clades of the *Ty3/gypsy* superfamily (bold lines) are included in the maximum likelihood tree construction. Black arrow head marks the position of *lennie* characterized in *Echinococcus granulosus*. The gray dotted box highlights *Saci2*-like elements of *S. erinaceieuropaei* selected for detailed characterization. Families diversified from the common *Saci2*-like ancestor are annotated as *Se_Saci2-1* to *-8.* Entries used for the determination of full-length retrotransposon sequences are indicated in bold. Support values are shown for major nodes.

BLASTp (ver. 2.15.0) search using the *Eg_lennie* Pol sequence identified a total of 1260 matches in the *S. erinaceieuropaei* proteome with various query coverage values (Max. target sequences = 5000; *E*-value < 1 × 10^−6^). Of these, 81 entries were finally selected to determine their phylogenetic positions against multiple representative LTR retrotransposons based on the RT-RH-IN sequence information (Figure 1). Most of the *Spirometra* Pol proteins were co-clustered with those in *Saci2*/*Eg_lennie* (21 entries) or *CsRn1* (59 entries) clades. The putative retrotransposons encoding the *Saci2*-like Pol proteins were collectively named *S. erinaceieuropaei_Saci2* (*Se_Saci2*), rather than ‘*lennie*’ to maintain the phylogenetic priority of *Saci2*, and applied in detailed characterizations.

### 2.2. Se_Saci2 Retrotransposons in S. erinaceieuropaei

A full-unit LTR retrotransposon encompassing the coding DNA sequence (CDS) of VZI35137.1 was identified in a *S. erinaceaieuropaei* genome contig (CACRXP010000411.1). The element possessed 5′- and 3′-LTRs starting with 5′-TGT-3′ and ending with 5′-ACA-3′, and was flanked by identical 4-bp target site duplications (TSD). Using this full-length sequence, paralogous sequences were further retrieved from their respective genomic loci through BLASTn search. Additional full-unit retrotransposons were identified in the same way using CDSs of other Pol proteins (bold in Figure 1). Identities of these mobile elements were verified by comparing their LTR sequences and clustering patterns in the phylogenetic tree. Finally, eight distinct *Saci2*-like retrotransposons were identified in the *S. erinaceieuropaei* genome and titled *Se_Saci2-1* to *-8* (Figure 1). The nucleotide sequences of each paralogous copy group revealed numerous base substitutions that had either sporadically occurred or been clonally inherited among them. Notably, a series of diagnostic base substitutions and indels in several *Se_Saci2-6* copies, designated *Se_Saci2-6a* sublineage, were clearly distinct from those in the rest, *Se_Saci2-6b* sublineage. The *Se_Saci2-6a* sublineage members formed a tight clade interconnected with shorter branches in phylogenetic trees based on LTR or full-unit sequences (Supplemental Figure S1).

**Figure 2:**
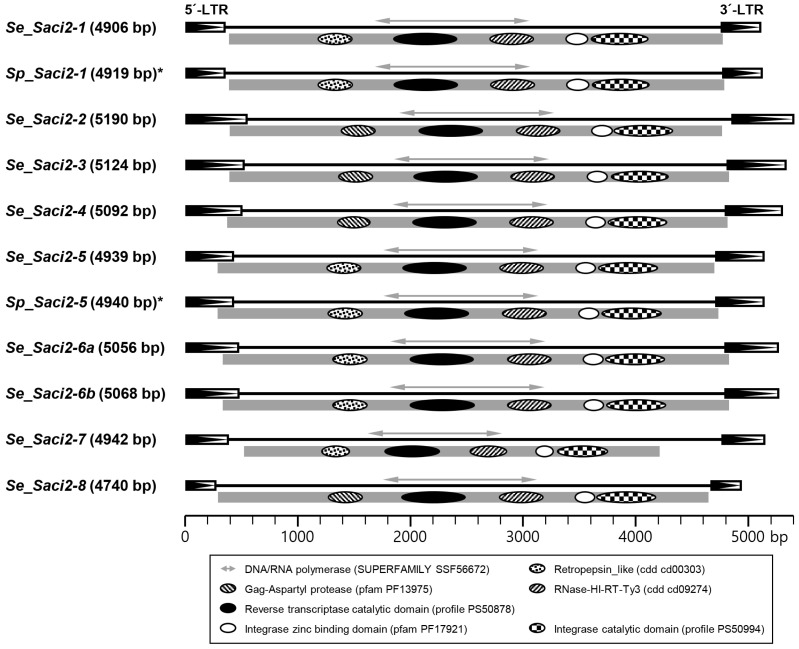
Overall structures of *Saci2-like* retrotransposon families identified in *Spirometra erinaceieuropaei* (*Se_Saci2*) and *Sparganum proliferum* (*Sp_Saci2*, asterisks). Consensus structures were reconstructed from paralogous copies. Black arrowhead boxes denote flanking long terminal repeats (LTRs). Grey bar indicates the open reading frame (ORF), predicted using ORF Finder. Conserved protein domains within the ORF are shown as variously filled ellipses (legend below). Bidirectional arrows mark regions corresponding to DNA/RNA polymerase domain sequences used for evolutionary analyses including the calculation of dN/dS ratios. Scale bar indicates nucleotide positions in bp.

Due to indels, *Se_Saci2* copies varied in size and frequently carried premature stop codons within their ORFs (Supplemental Table S1). Nevertheless, many LTR pairs showed > 99% identity, suggesting their relatively recent expansion in the genome. Consensus *Se_Saci2* elements encoded a Gag-Pol fusion protein (Figure 2), and one *Se_Saci2-6a* copy preserved an intact ORF nearly identical to the consensus (Supplemental Table S1). Amino acid sequences of these *Se_Saci2* polyproteins exhibited similarity values ranging from 37% to 72% to one another. All *Se_Saci2* elements were flanked by 4-bp TSDs, although no consensus sequence was observed among them.

### 2.3. Se_Saci2 Orthologs in Other Parasitic Cestodes

LTR retrotransposons orthologous to *Se_Saci2*, named *S. proliferum Saci2* (*Sp_Saci2*) were readily identified in the genome of *S. proliferum*, through BLAST searches (Supplemental Table S2). The structural integrity and coding potential of these *Sp_Saci2* copies were also severely disrupted by sporadic indels and base substitutions. Moreover, numbers of the isolated paralogous copies were much smaller than those of *Se_Saci2* families. As a result, consensus sequences only for *Sp_Saci2-1* and *-5* could be determined from sequence alignments (Figure 2 and Supplemental Table S2).

Additional orthologs were isolated from *L. intestinalis* (*Li_Saci2*) and *S. solidus* (isolate ULSS0001; *Ss_Saci2*), through BLAST searches using the internal regions of respective *Se_Saci2* elements (query coverage > 70%, Supplemental Table S3). During sequence retrieval, copies located on genomic contigs shorter than 10 kilobases were excluded to avoid chimeric sequences potentially resulting from shotgun fragment assembly. Nucleotide sequences of all the *Se_Saci2* homologs were aligned with one another, to prepare alignment sets for next-step phylogenetic analyses (Int and RT regions; Figure 2 and Supplemental Table S3).

### 2.4. Evolutionary Statistics Among Cestode Saci2 Element Copies

**Figure 3:**
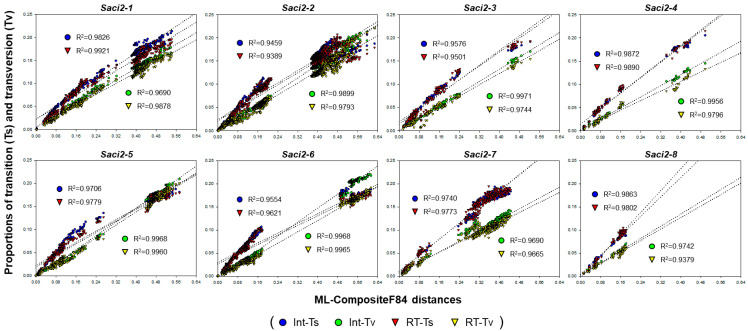
Substitution saturation plots of *Saci2*-like homologs in diphyllobothriidean genomes. Proportions of transition (Ts; blue and red) and transversion (Tv; green and yellow) are plotted against maximum-likelihood distances for Int (circles) and RT (tringles) sequences. Correlation coefficients (R^2^) obtained from linear regression of each dataset (dotted lines) are shown within the graphs.

Prior to phylogenetic analyses, the Int and RT sequence alignments were evaluated with the Xia’s test to assess substitution saturation. The observed Iss values (0.2314–0.4435) were significantly lower than critical values for both symmetrical and asymmetrical topologies (*p* < 0.0001), indicating that base substitutions were not saturated, even at intergenomic levels, and thus suitable for phylogenetic inference (Table 1). Consistently, proportions of transition (Ts) and transversion (Tv) correlated linearly with genetic distances. Despite these strong linear correlations, however, the plots showed a bimodal distribution pattern with a gap between lower (in paralogous copy pairs) and higher (in orthologous copy pairs) distance ranges. (Figure 3). The proportions increased linearly in the lower range, but began to plateau at higher distances, suggesting onset of weak saturation among orthologous copies. Similar results were obtained from the full-unit *Se_Saci2* paralogs (Supplemental Tables S1 and S4; Supplemental Figure S2).

To further assess potential recombination effects, divergence was examined in sliding windows across aligned *Saci2* RT sequences (Supplemental Figure S3). Divergence levels were relatively uniform across the entire sequences, without localized peaks that would indicate recombination tracts. The uniform divergence rate may suggest that, if homologous recombination occurred, it mainly involved copies of similar age or evolutionary status. Therefore, the overall evolutionary statistics remain robust at the population level and are not significantly affected by recombination.

**Figure 4:**
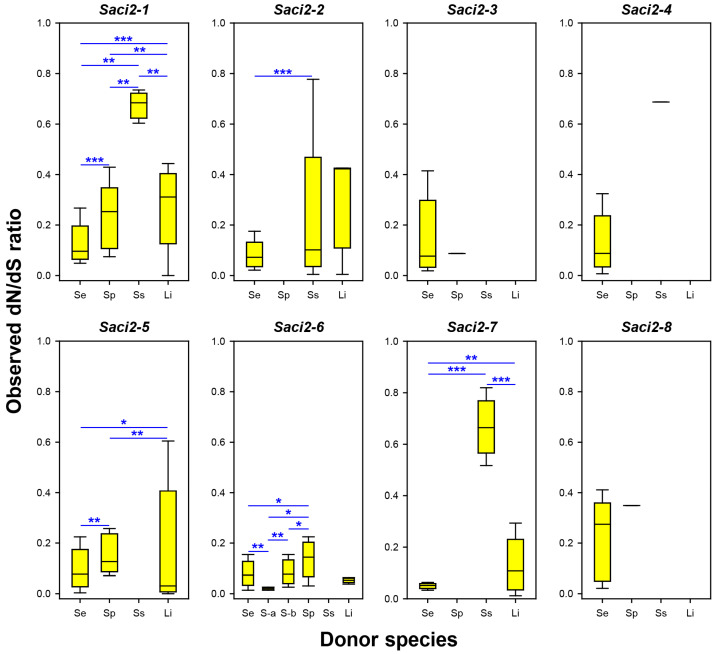
Observed dN/dS ratios for DNA/RNA polymerase domain sequences of *Saci2* families in diphyllobothriidean genomes. Box plots show distribution of the dN/dS ratios among paralogous copies of each family (*Saci2-1* to *-8*), grouped by donor species: Se, *Spirometra erinaceieuropaei*; Sp, *Sparganum proliferum*; Ss, *Schistocephalus solidus*; and Li, *Ligula intestinalis*. Sublineages *Se_Saci2-6a* (S-a) and *Se_Saci2-6b* (S-b) are presented separately. Yellow boxes represent the interquartile range (Q1–Q3), with horizontal lines indicating the median. Whiskers extend to the minimum and maximum observed values. Statistical significance of differences was assessed by the Mann–Whitney U test. *p* values: *, 0.05–0.01; **, 0.01–0.001; and ***, < 0.001.

The sequence alignments were further subjected to the Tajima’s D, and Fu and Li’s D and F tests (Table 2). The Int regions of most *Saci2* families showed negative values, often with statistical significance (*p* < 0.05), though the results varied by donor and family. For instance, all parameters obtained from the *Se_Saci2-1* copies were highly significant, whereas those from the *Sp_Saci2-1* and *Li_Saci2-1* copies were not. Among orthologs, only the *Li_Saci2-5* and *-7* copies showed the signs of strong purifying selection. Similar trends were observed for RT regions, with generally larger absolute values. These observations showed an excess of low-frequency variants in the cestode *Saci2* copies, reflecting purifying selection and/or recent expansions through the error-prone retrotransposition mechanism. To examine the probable selection pressure, dN/dS ratios were estimated by directly counting codon changes, rather than applying model-based approaches, due to their heavily disrupted ORFs (Figure 4). Median dN/dS ratios ranged from 0.051 (*Se_Saci2-7*) to 0.422 (*Li_Saci2-2*) across *S. erinaceieuropaei*, *S. proliferum*, and *L. intestinalis*, with particularly low ratios in *Se_Saci2-6a* subgroup (median 0.021). In contrast, the ratios were markedly elevated among the *Ss_Saci2-1* (0.684), *-4* (0.687), and *-7* (0.665) copies, indicating relaxed constraints. Furthermore, the dN/dS ratios differed significantly between several paralogous groups (*p* < 0.05; asterisks in Figure 4). Collectively, most cestode *Saci2* elements appeared to be under varying degrees of purifying selection depending on family and host genome (Supplemental Figure S4).

### 2.5. Phylogenies of Cestode Saci2 Elements

As expected under differential selection pressures, diversity values varied considerably among *Saci2* families in relation to their donor species. To better assess their evolutionary status in host genomes, p-distances were calculated from the *Saci2* Int sequences (Table 3). The overall values ranged from 0.137 (*Saci2-8*) to 0.415 (*Saci2-5*), with broad intragenomic variation: 0.026–0.123 in *S. erinaceieuropaei*, 0.114–0.170 in *S. proliferum*, 0.186–0.585 in *S. solidus*, and 0.031–0.544 in *L. intestinalis*. Orthologous pairs showed much higher distances, reaching up to 0. 932 between *Ss_Saci2-6* and *Li_Saci2-6*. RT-based calculations revealed similar distribution patterns across paralogous and orthologous pairs, although the values themselves were slightly lower than those from Int sequences (Table 3). Within the *Se_Saci2-6* sublineages, intragenomic Int distances differed markedly (0.019, 0.072, and 0.090 for *Se_Saci2-6a, -6b*, and *-6*, respectively), indicating that the *Se_Saci2-6a* lineage most recently diverged from the parental *Se_Saci2-6b.* Nonetheless, all the three groups showed comparable distances to orthologous *Saci2-6*.

**Figure 5:**
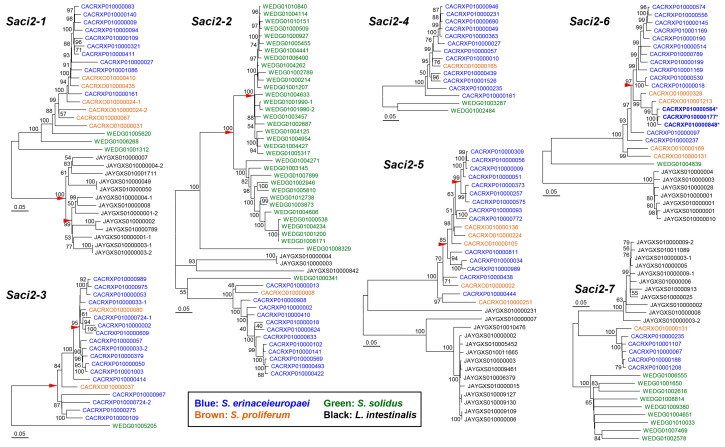
Phylogenetic relationships of *Saci2* families in diphyllobothriidean genomes. Maximum likelihood trees based on the internal-region sequences of *Saci2* ortholog groups. Sequence identities are given by the GenBank accession numbers of relevant genomic contigs, color-coded by species: blue, *Spirometra erinaceieuropaei*; brown, *Sparganum proliferum*; green, *Schistocephalus solidus*; and black, *Ligula intestinalis.* Red arrowheads denote diversification points of sublineages. The *Se_Saci2-6a* copies are shown in bold. Numerals at the branching nodes indicate bootstrap support from 1000 replicates.

Most paralogous *Saci2* groups were well separated in Int sequence-based phylogenetic trees along with the phylogeny of their donor species, although the genetic distances among them highly varied (Figure 5). A significant fraction of these *Saci2* copies appeared monophyletic, demonstrating they propagated from a single master lineage. Some of the monophyletic groups further branched into paraphyletic subgroups (red arrowheads in Figure 5). The branching topology may indicate that a novel master lineage(s) of the element had emerged in the relevant genome either for transiently overlapping or sequential succeeding replication of its progeny. Notably, none of the *Sp_Saci2* elements formed a distinct monophyletic group(s) in the maximum-likelihood trees. Given the small number of nearly intact *Sp_Saci2* elements and the extensive disruption within their ORFs, this branching pattern suggests that *Saci2* elements have lost their mobility and entered a decline phase in the *S. proliferum* genome during relatively recent evolutionary time.

## 3. Discussion

As an intragenomic retroviral elements, autonomous retrotransposons replicate by inserting progeny copies into new genomic loci through the sequential action of host RNA polymerase II and their own RT [9]. Once integrated, these copies are subject to selection pressures across two distinct dimensions: (1) the fate of the retrotransposon copy-occupied loci and (2) the divergence of the copy sequences. Since transposition events increase genomic dosage or disrupt nearby genes via insertional mutagenesis and ectopic recombination, most new insertions are ultimately eliminated from the genome. However, if an insertion is beneficial or at least neutral to host survival, it may persist through genetic drift or natural selection [18,19]. Retrotransposon copies maintained in the genome diverge in sequence from their parental types due to replication errors, inefficient DNA repair, or error-prone enzyme activity during propagation [20,21]. Historically treated as non-coding genomic segments, even when coding potential is retained, they are presumed to evolve under relaxed or neutral selection, allowing rapid accumulation of base substitutions [22,23]. Over time, most retrotransposon copies lose mobility through deleterious mutations, and only a limited number that retain intact ORFs and *cis*-regulating motifs propagate as active masters [24,25]. The dominant lineage periodically shifts, driven by the stochastic emergence and expansion of new master copies [26]. Few studies have directly compared selection pressures on active versus inactive TE lineages. Thus, it remains uncertain whether both types can be validly co-analyzed using a single model, given their distinct selective regimes [21,27]. To reduce discrepancies between active and inactive lineages, and between coding and non-coding regions, this study focused on ORF sequences from recently expanded retrotransposon copies.

LTR retrotransposons in cyclophyllideans with small genome sizes show a narrow taxonomic distribution. These elements are mainly assigned to the most predominant clades: *Saci2* and *CsRn1*. Despite their abundance, only a consensus sequence for a single *Saci2*-like element (*lennie*) has been determined in both *E. granulosus*, *E. multilocularis*, and *T. solium* genomes, due to the prevalence of structurally impaired copies in these cyclophyllidean genomes [16,17]. *Saci2* and *CsRn1* homologs are also most plentiful in the *S. erinaceieuropaei* genome, in reversed abundance order compared to cyclophyllideans (Figure 1). The *Saci2* clade of *S. erinaceieuropaei* comprises eight distinct families (*Se_Saci2-1* to *Se_Saci2-8*). Multiple *Se_Saci2* copies preserve complete or near-complete structures, and remarkably, at least one copy has retained an intact ORF identical to that predicted from a consensus sequence (Figure 2 and Supplemental Table S1). Given the high identity values between flanking LTRs, these findings suggest that the *Se_Saci2* elements are likely still actively propagating in the *S. erinaceieuropaei* genome. This contrasts sharply with their cyclophyllidean homologs, which appear to have entered decline phases in recent evolutionary time. Homologous elements are also identified in *S. solidus* and *L. intestinalis*. Diphyllobothriidean *Saci2*-like elements, rather than the more abundant *CsRn1* homologs, were selected in this study to compare their genomic and evolutionary status with cyclophyllidean homologs. Furthermore, their well-preserved structures and distinct divergence patterns make them ideal for comparative analyses of retrotransposon dynamics and genome size evolution between the two lineages.

Despite maintaining overall structure and coding profile, consistent with descent from a last common ancestor, the *Se_Saci2* elements exhibited considerable divergence in their nucleotide sequences (40–64% similarities based on consensus versions) and lengths (4740–5190 bp). These values fall below the commonly applied 70% threshold for retrotransposon family designation, supporting the classification of *Se_Saci2-1* to *-8* as distinct families. The observed polymorphisms were largely attributable to their flanking LTRs (15–40% similarities and their 251–521 bp in length, respectively; Figure 1 and Supplemental Table S1). A similar divergence pattern was observed in the *Se_Saci2-6a* lineage, which had diverged from the parental *Se_Saci2-6b* lineage. This was evidenced by differences in their consensus sequence similarities (88% for full sequences vs. 77% for LTR sequences between *Se_Saci2-6a* and *Se_Saci2-6b*, respectively) and 5′-LTR length (449 bp vs. 452 bp; Supplemental Table S1 and Supplemental Figure S1). Average genetic distances between near-complete paralogous copies of the two *Se_Saci2-6* sublineages shown in Supplemental Table S1 were 0.173 ± 0.007 and 0.392 ± 0.046 for their full and 5′-LTR sequences, respectively. These values were significantly larger than those at intra-lineage levels (0.022 ± 0.002 and 0.034 ± 0.007 for *Se_Saci2-6a* lineage; 0.067 ± 0.002 and 0.096 ± 0.007 for *Se_Saci2-6b* lineage; Supplemental Figure S5). These findings may suggest that a change in the LTR region facilitates the emergence of novel families from pre-existing retrotransposons. Base substitutions caused by error-prone replication of the retroelement genomes and other genetic events such as homologous and/or non-homologous recombination contribute to the generation of diversified LTRs. Beyond their role as structural boundaries, LTR regions of retrotransposons act as crucial *cis*-regulatory elements, including promoters and enhancers [28,29]. These regulatory functions allow LTRs to precisely control the retrotransposon’s life cycle within host genome primarily by regulating generation of its mRNA transcripts—a key step for replication and transposition [8,30]. LTRs also define both the transcription initiation and termination sites, which is essential for maintaining the structural integrity of retrotransposons [31,32]. Consequently, even minor alterations within LTR sequences can significantly influence transposition frequency of concerned retrotransposon by changing the relative strength of these regulatory elements, and even leading to changes in its identity, as described above [33]. Meanwhile, LTR retrotransposon utilizes the host’s tRNA molecule as a primer for the synthesis of the first complementary DNA strand by endogenous RT [8]. Base substitutions in primer binding site (PBS) of retrotransposons can either modulate the binding affinity of the corresponding tRNA molecule or alter the primer tRNA molecules with different stoichiometric numbers (Supplemental Figure S6), both of which affect the element’s transposition rate by influencing the initiation of first-strand cDNA synthesis. These tight correlations between LTR/PBS sequences and retrotransposon’s mobile activity are therefore strong determinants in regulating its copy number within the host [31,34,35], as seen in the *Saci2* families across cestode species (Supplemental Tables S1–S3).

Retrotransposons are often concentrated in heterochromatic regions such as centromeres and telomeres [36], which are generally underrepresented in draft genome assemblies due to their repetitive nature [37]. As a result, the retrotransposon copies identified in current cestode genomes seem to correspond to those residing in euchromatic regions. Given that transcription is highly restricted within tightly packed heterochromatin [38], these euchromatic copies are more likely to reflect the true evolutionary dynamics and mobilization potential of retrotransposon families. Paralogous copies of *Saci2* families with > 90% of their consensus lengths show marked differences in copy number and genetic divergence across *S. erinaceieuropaei*, *S. proliferum*, and related diphyllobothriidean cestodes (Table 3 and Supplemental Tables S1–S3). This suggests that *Saci2* elements have followed family-specific evolutionary trajectories, shaped by a balance of intrinsic and extrinsic factors [39,40,41,42,43]. While host defense mechanisms presumably act with similar intensity across *Saci2* families derived from a common ancestor, intrinsic factors—such as promoter/enhancer strength and primer utilization efficiency, both of which are profoundly affected by base substitutions in respective regions—may account for their differential evolutionary outcomes. Indeed, base substitutions in the PBS regions appear to have modified the set of tRNA primers used by each family (Supplemental Figure S6). The copy numbers of genes encoding the relevant tRNAs are estimated to be similar in *S. erinaceieuropaei* (e.g., 25 for tRNA^Leu^_AAG, 24 for tRNA^Gly^_GCC, and 28 for tRNA^Lys^_CTT; my unpublished data). Although these values are based on a draft genome assembly, they fall within the range observed in other flatworm and eukaryotic genomes, suggesting that they are not markedly inflated by potential assembly redundancy [44]. Competition for shared tRNAs or differences in annealing affinity caused by base mispairing may influence the efficiency of reverse transcription [45,46]. Future work should investigate additional element-intrinsic factors, including variation in LTR regulatory motifs and the role of small RNA-mediated silencing, which remains largely elusive in cestodes.

Base substitutions in the internal regions of the *Saci2* families appeared not to be saturated among paralogous pairs, but signs of mild saturation were detected among orthologous pairs (Table 1 and Figure 3). The incipient plateau in the transition proportions relative to genetic distances among orthologous pairs, especially in *Saci2-3*, *-5*, and *-6*, suggested that base substitutions are at an early stage of saturation at the orthologous level (Figure 3). Accordingly, maximum likelihood phylogenetic analyses shown in Figure 5 were conducted under the GTR + G + I model, which is statistically consistent and robust for datasets exhibiting mild substitution saturation, due to its ability to accommodate rate heterogeneity and invariant sites [47]. Tree topologies inferred under the Jukes–Cantor model were broadly consistent with those under the GTR model, further supporting that substitution saturation, if present, is minimal. Additionally, neutrality tests yielded negative values for all the family group sequences, although many were not statistically significant (Table 2). In general, significant negative values in neutrality tests indicate that minor alleles with rare base substitutions are selectively maintained under purifying selection due to their beneficial effects on the donors [48,49]. However, in the case of retrotransposons, these statistics should be interpreted with caution, since their underlying assumptions of allelic variation in panmictic populations do not apply. The negative values observed here are more likely attributable to recent bursts of retrotransposon amplification than to classical purifying selection, consistent with propagation through error-prone retrotransposition rather than Mendelian inheritance. Therefore, neutrality tests were treated as auxiliary indicators of skewed frequency distributions, whereas dN/dS ratios provide a more appropriate measure of selective pressure on retrotransposon coding regions. Taken together, phylogenetic inferences, along with the results from the saturation and neutrality tests, clearly demonstrate that *Saci2* families have undergone recent expansions in diphyllobothriidean genomes, with family- and host-specific patterns (Figure 5).

Retrotransposons are often assumed to evolve neutrally, due to their non-coding nature [50]. Coupled with weaker selective constraints, the error-prone retrotransposition process accelerates divergence among homologous copies. However, as mentioned above, genetic distances among LTR sequences were higher than those among ORF sequences in *Saci2* families. This observation suggests that functional constraints in coding regions impose purifying selection to enable long-term survival in the host genome [40,51,52,53]. In this context, the lower dN/dS ratios observed in the *pol* sequences of *Saci2* families offer compelling evidence for ongoing purifying selection. Although recent expansion reduces overall divergence, it does not inherently lower the dN/dS ratio, which tends to approach 1 without additional directional forces. Therefore, the consistently low dN/dS values observed here are best explained by purifying selection acting during or after expansion. In human HERV-K(HML2), recurrent infection by exogenous counterparts has been proposed as a selective force maintaining low dN/dS ratios [54]. However, such replacement by exogenous counterparts is unlikely in the *Saci2* families, which lack the *env* gene required for reinfection. Given the intrinsic accumulation of base substitutions, only a subset of copies stochastically retains intact ORFs and continues to propagate, while others gradually degrade into inactive genomic fragments. In this study, intact or near-intact *Saci2* family copies, which mostly reflect ongoing evolutionary dynamics, were selected for dN/dS analysis. Therefore, the lower dN/dS ratios observed in these sequences likely reveal purifying selection acting on functionally competent master lineages during their expansion. Additionally, DNA repair mechanisms such as mismatch repair and transcription-coupled repair, which are biased toward actively transcribed regions [55,56,57], may reinforce this selection. While these systems do not distinguish between synonymous and nonsynonymous mutations [58], they can increase the number of autonomous copies by reducing the overall mutation load in coding regions. The higher dN/dS ratios and larger genetic distances observed in the *S. solidus Saci2-1* and *-7* copies, which apparently represent a post-proliferative or quiescent state based on their connection by long branches, support this interpretation (Figure 4 and Figure 5; Table 3). Overall, the variation in dN/dS ratios among *Saci2* families likely demonstrates distinct balancing points between purifying selection and relaxed constraint, which shape the unique evolutionary landscapes of these families within their respective host genomes.

The *Saci2*- and *CsRn1*-like elements are dominantly present in cyclophyllidean and diphyllobothriidean genomes, although their genomic landscapes differ between the two orders (Figure 1 and [16])**.** These LTR retrotransposons have lost mobility and persist as fragmented remnants in modern cyclophyllideans, whereas they are actively or recently expanding under purifying selection in diphyllobothriideans. Furthermore, they have diversified into at least eight (*Saci2* homolog) and four (*CsRn1* homolog; unpublished data) with mobilization potentials varying by family and/or host, likely due to accumulated base substitutions, particularly in the LTRs. The pronounced reduction of retrotransposon populations and genome sizes in cyclophyllideans is unusual among multicellular eukaryotes. During their divergence from diphyllobothriideans, cyclophyllideans acquired traits such as simplified life cycles, greater developmental plasticity, accelerated replication, and increased dependence on host-derived nutrition, which may have favored genome streamlining by removing non-essential sequences. In this context, retrotransposons, abundant yet dispensable, would have been prime targets for elimination. Extensive inactivation and removal of these ‘non-coding intragenomic parasites’ likely reflect purifying selection to reduce genomic burden and improve adaptation to host-specific niches. Given the conserved nature of host defense systems probably responsible for the retrotransposon regulation [59,60] and the stochastic maintenance of some active retrotransposon copies, this widespread silencing cannot be easily attributed to host-driven or intrinsic mechanisms alone. Notably, distinct evolutionary dynamics are evident even between the closely related diphyllobothriideans, *S. erinaceieuropaei* and *S. proliferum*. In the non-proliferative *S. erinaceieuropaei*, *Saci2* families tend to retain structural integrity and higher mobilization potential, whereas in the proliferative *S. proliferum*, certain families show reduced activity and greater sequence divergence. This difference may relate to life-history strategy: extensive asexual reproduction in *S. proliferum* could reduce the selective advantage of maintaining large populations of active retrotransposons, shifting the balance toward gradual inactivation. Further investigations are needed to uncover the biological or molecular triggers responsible for retrotransposon inactivation and to address broader principles of genome architecture and TE control across metazoans.

## 4. Materials and Methods

### 4.1. Retrieval of Amino Acid Sequences Homologous to the Pol Protein of E. granulosus Lennie

The proteomic database of *S. erinaceieuropaei* in the GenBank (Japanese isolate [6]) was surveyed with the amino acid sequence of *lennie* Pol identified in *E. granulosus* (1327 aa) using the BLASTp program (ver. 2.15.0). The amino acid sequences of *Spirometra* proteins retrieved during the homology search were aligned with those of other LTR retrotransposons representing each of the distinct clades of the *Ty3/gypsy* family [61] using the MUSCLE program (ver. 5.0). After extracting columns containing sequences corresponding to the reverse transcriptase (RT)-RNase H (RH)-integrase (IN) domains, the alignment was used as an initial input in a phylogenetic analysis to construct a maximum likelihood tree using the PhyML program (ver. 3.1; Jones-Taylor-Thornton model for amino acid evolution, gamma distributed rates among sites, and pairwise deletion of missing data [62]). Statistical support of each branching node was inferred using the non-parametric Shimodaira-Hasegawa-like approximate likelihood ratio test (SH-aLRT) and the resulting tree was displayed by TreeView.

### 4.2. Identification of Full-Unit LTR Retrotransposons in S. erinaceieuropaei Genome

DNA sequences (approximately 15 kb) encompassing CDSs of the *Spirometra* Pol proteins were extracted from their respective genomic contigs by BLASTn searches using the CDSs as queries (Japanese isolate, Assembly SerJ_v2_0 [6]). Common genetic elements were identified by comparing multiple matching sequences, as described previously [16]. Structural boundaries of these repetitive elements were confirmed by recognizing two target site duplications (TSDs), if possible, at direct upstream and downstream regions of the 5′- and 3′-LTRs, respectively. The nucleotide sequences of multiple retrievals against a single query were aligned and a consensus sequence of full-unit retrotransposon was derived from the alignment using the GeneDoc program (ver. 2.7.000). The consensus sequence was used in the prediction of open reading frame (ORF) with ORF Finder (https://www.ncbi.nlm.nih.gov/orffinder/, accessed on 20 August 2025), as well as in the determination of architectural characters. Protein domains conserved in the theoretical amino acid sequences were analyzed using the Simple Modular Architecture Research Tool (SMART; ver. 8.8; https://smart.embl.de/, accessed on 20 August 2025) and InterProScan (ver. 5.55-88.0; https://www.ebi.ac.uk/interpro/search/sequence/, accessed on 10 May 2022) programs.

Complete nucleotide sequences of the consensus retrotransposons were used as queries in the screening of the *S. erinaceieutopaei* and *S. proliferum* (Venezuelan isolate, Assembly SprV_v2_2 [6]) genomes in GenBank using BLASTn to retrieve their paralogous and orthologous copies, respectively (megablast algorithm; cutoff for query coverage, 70 or 90%). Whole genome shot-gun (WGS) contigs in the sequence data bank were also searched for the isolation of orthologous sequences from other cestode genomes using the consensus internal-region sequences of the *Spirometra* elements (blastn algorithm; cutoff for query coverage, 70%; *E*-value < 1 × 10^−5^). In this study, the terms “paralogous” and “orthologous” are used in a pragmatic sense to indicate homologous retrotransposon copies within the same donor species and across different donor species, respectively, since positional conservation or shared synteny is generally not applicable to mobile genetic elements.

### 4.3. Sequence Analyses

Daughter copies of each retrotransposon, retrieved through BLASTn searches, were aligned with one another. After removing copy- or species-specific insertions/deletions (indels), the sequence alignments were analyzed using the Data Analysis in Molecular Biology and Evolution (DAMBE, ver. 7.3.32 [63]). The Xia’s test was performed to calculate the entropy-based index of substitution saturation (Iss) along with critical (Iss.c) values under assumptions of symmetrical and extremely asymmetrical tree topologies. The proportions of transition (Ts) or transversion (Tv) across the entire alignment were plotted against corrected genetic distances, calculated under the ML-CompositeF84 substitution model. To investigate the probable excess of rare copy(s), the alignments were also subjected to Fu an Li’s test and Tajima’s test using DNA Sequence Polymorphism (DnaSP, ver. 6.12.03 [64]). Nucleotide sequences corresponding to CDS of DNA/RNA polymerase domain (SUPERFAMILY entry no. SSF56672; 1311 bp) were extracted from the cestode *Saci2* alignments. The numbers of synonymous (dS) and non-synonymous (dN) codon changes were empirically counted from the codon-based alignments at both intra- and intergenomic levels, using a simplified counting approach that was conceptually similar to that of Nei and Gojobori [65]. During this analysis, gaps and stop codons introduced by indels and/or base substitutions were removed as missing data in a pairwise manner, and the resulting counts were not corrected for multiple substitutions or estimation of synonymous and non-synonymous sites. Significance of differences in dN/dS ratios among intragenomic paralogous groups was statistically tested using Mann–Whitney U test (*p* < 0.05).

### 4.4. Phylogenetic Analyses

Nucleotide sequences corresponding to internal (Int) and DNA/RNA polymerase domain (RT) regions (Supplemental Table S3) were aligned using ClustalW (https://www.genome.jp/tools-bin/clustalw, accessed on 29 April 2025). The resulting alignments were manually refined with GeneDoc, guided by codon positions predicted through translation using the TRANSLATE tool in the Sequence Manipulation Suite (https://www.bioinformatics.org/sms2/translate.html, accessed on 1 May 2025), to ensure accuracy at the amino acid sequence level. Based on these alignments, mean p-distances (proportions of different sites) were calculated within and between paralogous sequence groups using MEGA 11, employing the maximum composite likelihood model with a homogeneous gamma distribution for rate variation among sites. During these calculations, positions with less than 90% site coverage were eliminated, while alignment gaps fewer than 10% were allowed at any position. Standard errors were estimated with 1000 bootstrap replicates. The Int alignments were also used in the construction of maximum likelihood trees with General Time Reversible model for base substitutions and a gamma distribution with invariant sites (GTR + G + I). Gaps in the alignment were partially deleted during distance calculation for the tree construction (site coverage cutoff, 90%). Branching support was assessed through bootstrap analysis with 1000 replicates of the initial input.

## 5. Conclusions

*Saci2*- and *CsRn1*-like elements dominate in both cyclophyllidean and diphyllobothriidean genomes, but their evolutionary trajectories differ. In cyclophyllideans, they persist mainly as fragmented remnants, whereas in diphyllobothriideans they remain active or have recently expanded under purifying selection. The reduction of retrotransposons and genome size in cyclophyllideans likely reflects genome streamlining linked to simplified life cycles and accelerated replication. Within diphyllobothriideans, *S. erinaceieuropaei* retains structurally intact and mobilization-competent families, while *S. proliferum* shows early signs of retrotransposon inactivation. This inactivation may be related to its distinctive trait of extensive asexual reproduction, and future investigations into this link will help clarify the interplay between life-history strategies and TE control in cestodes.

## Figures and Tables

**Table 1:** Xia’s saturation test for base substitutions in cestode *Saci2* element copies.

Alignment	Donor Organism (Number of Sequences) ^a^	Iss	95% Confidence Interval	Iss.cSym	*P*_1_ ^b^	Iss.cAsym	*P*_2_ ^b^
*Saci2-1*	**All cesdtodes examined (32)**	**0.4122**	**0.3962–0.4282**	**0.8091**	**0.0000**	**0.5562**	**0.0000**
	*Spirometra erinaceieuropaei* (10)	0.1102	0.1010–0.1194	0.8306	0.0000	0.7236	0.0000
	*Sparganum proliferum* (6)	0.1566	0.1448–0.1684	0.8377	0.0000	0.7877	0.0000
	*Ligula intestinalis* (13)	0.3531	0.3359–0.3702	0.8250	0.0000	0.6895	0.0000
*Saci2-2*	**All cesdtodes examined (51)**	**0.3602**	**0.3415–0.3790**	**0.8044**	**0.0000**	**0.5426**	**0.0000**
	*S. erinaceieuropaei* (12)	0.1700	0.1579–0.1822	0.8192	0.0000	0.6855	0.0000
	*Schistocephalus solidus* (35)	0.2354	0.2207–0.2500	0.8077	0.0000	0.6181	0.0000
*Saci2-3*	**All cesdtodes examined (20)**	**0.2729**	**0.2594–0.2863**	**0.8086**	**0.0000**	**0.6235**	**0.0000**
	*S. erinaceieuropaei* (17)	0.2517	0.2383–0.2562	0.8118	0.0000	0.6419	0.0000
*Saci2-4*	**All cesdtodes examined (15)**	**0.3016**	**0.2874–0.3158**	**0.8183**	**0.0000**	**0.6643**	**0.0000**
	*S. erinaceieuropaei* (12)	0.1880	0.1753–0.2006	0.8233	0.0000	0.6927	0.0000
*Saci2-5*	**All cesdtodes examined (33)**	**0.3673**	**0.3477–0.3869**	**0.8040**	**0.0000**	**0.5419**	**0.0000**
	*S. erinaceieuropaei* (14)	0.2858	0.2691–0.3025	0.8154	0.0000	0.1596	0.0000
	*S. proliferum* (5)	0.1392	0.1263–0.1522	0.8355	0.0000	0.7982	0.0000
	*L. intestinalis* (14)	0.1514	0.1481–0.1711	0.8170	0.0000	0.6742	0.0000
*Saci2-6*	**All cesdtodes examined (28)**	**0.3332**	**0.3157–0.3507**	**0.8028**	**0.0000**	**0.5879**	**0.0000**
	*S. erinaceieuropaei* (16) ^c^	0.1550	0.1435-0.1666	0.8274	0.0000	0.6696	0.0000
	*S. erinaceieuropaei*_b (13)	0.1543	0.1414–0.1673	0.8246	0.0000	0.6886	0.0000
	*L. intestinalis* (7)	0.1146	0.1039–0.1253	0.8356	0.0000	0.7686	0.0000
*Saci2-7*	**All cesdtodes examined (26)**	**0.4435**	**0.4248–0.4622**	**0.8034**	**0.0000**	**0.5908**	**0.0000**
	*S. erinaceieuropaei* (5)	0.2558	0.2352–0.2763	0.8362	0.0000	0.7998	0.0000
	*S. solidus* (9)	0.4214	0.4026–0.4402	0.8260	0.0000	0.7260	0.0000
	*L. intestinalis* (11)	0.1701	0.1568–0.1835	0.8219	0.0000	0.6987	0.0000
*Saci2-8*	**All cesdtodes examined (12)**	**0.2314**	**0.2172–0.2455**	**0.8205**	**0.0000**	**0.6877**	**0.0000**
	*S. erinaceieuropaei* (10)	0.2278	0.2135–0.2422	0.8243	0.0000	0.7123	0.0000

^a^ Saturation status of copies with paralogous numbers greater than five was estimated at the intragenomic level (see also Supplemental Table S1). ^b^ The statistical significance of differences between Iss and Iss.cSym (*P*_1_), or Iss and Iss.cAsym (*P*_2_), was examined using a two-tailed Student’s *t*-test. ^c^ *S. erinaceieuropaei* (16) includes both the *Se_Saci2-6a* and *Se-Saci2-6b* elements, whereas *S. erinaceieuropaei*-b (13) includes only the *Se-Saci2-6b* element of *S*. *erinaceieuropaei.*

**Table 2:** Tests for evolutionary modes of cestode *Saci2* elements.

Alignment	Donor Organism (Number of Squences) ^a^	Int Region			RT Region		
		Tajima’s D	Fu and Li’s D	Fu and Li’s F	Tajima’s D	Fu and Li’s D	Fu and Li’s F
*Saci2-1*	**All cesdtodes examined (32)**	−1.31144 *	−1.73527 *,^b^	−1.46136 *	−1.40987 *	−1.85638 *	−2.02127 **
	*Spirometra erinaceieuropaei (10)*	−1.92425 ***	−2.17508 ***	−2.38869 ***	−1.94333 ***	−2.23464 ****	−2.44426 ****
	*Sparganum proliferum* (6)	−1.09044 *	−1.02791 *	−1.14655 *	−1.22400 *	−1.17210 *	−1.30227 *
	*Ligula intestinalis* (13)	−1.29726 *	−0.93173 *	−1.18111 *	−1.29678*	−0.90270 *	−1.15661 *
*Saci2-2*	**All cesdtodes examined (51)**	−1.20565 *	−1.68992 *	−1.80102 *	−1.27304 *	−1.88385 **	−1.97508 **
	*S. erinaceieuropaei* (12)	−1.37539 *	−1.64454 *	−1.79592 *	−1.34268 *	−1.71113 *	−1.84185 *
	*Schistocephalus solidus* (35)	−1.84749 ***	−2.86640 ***	−2.98393 ***	−1.88691 ***	−3.03760 ***	−3.13260 ***
*Saci2-3*	**All cesdtodes examined (20)**	−1.87764 ***	−2.43667 ***	−2.64791 ***	−1.84227 ***	−2.34903 **	−2.56387 **
	*S. erinaceieuropaei* (17)	−1.61478 **	−2.02830 **	−2.21366 **	−1.65100 **	−2.07445 **	−2.26358 **
*Saci2-4*	**All cesdtodes examined (15)**	−1.79439 ***	−1.84293*	−2.11068 **	−1.78919 **	−1.83971 *	−2.10621 **
	*S. erinaceieuropaei* (12)	−1.78627 ***	−2.17931 ***	−2.36896 ***	−1.78175 ***	−2.17335 ***	−2.36249 ***
*Saci2-5*	**All cesdtodes examined (33)**	−0.68779 *	−1.80233 *	−1.68241 *	−0.80231 *	−1.99026 **	−1.87602 *
	*S. erinaceieuropaei* (14)	−1.29237 *	−1.60660 *	−1.74782 *	−1.24387 *	−1.64771 *	−1.76641 *
	*S. proliferum* (5)	−0.87003 *	−0.80076 *	−0.88873 *	−0.93071 *	−0.88925 *	−0.97847 *
	*L. intestinalis* (14)	−2.16686 ****	−2.49157 ***	−2.76247 ****	−2.15851 ****	−2.48925 ***	−2.75779 ****
*Saci2-6*	**All cesdtodes examined (28)**	−0.66645 *	−1.24721 *	−1.24641 *	−0.73956 *	−1.46510 *	−1.44722 *
	*S. erinaceieuropaei* (16) ^c^	−1.40545 *	−1.61597 *	−1.80025 *	−1.52392 *	−1.79126 *	−1.98406 *
	*S. erinaceieuropaei*_b (13)	−1.60743 **	−1,80309 *	−2.00568 *	−1.69063 **	−1.93048 **	−2.13776 **
	*L. intestinalis* (7)	−1.27810 *	−1.34293 *	−1.47382 *	−1.40954 *	−1.51122 *	−1.65026 *
*Saci2-7*	**All cesdtodes examined (26)**	−1.11942 *	−1.69876 *	−1.78098 *	−1.15422 *	−1.73427 *	−1.82259 *
	*S. erinaceieuropaei* (5)	−0.93593 *	−0.93593 *	−1.01835 *	−1.00076 *	−1.00076 *	−1.08369 *
	*S. solidus* (9)	−1.42583 *	−1.23910 *	−1.44474 *	−1.41018 *	−1.23146 *	−1.43390 *
	*L. intestinalis* (11)	−1.92872 ***	−2.21988 ***	−2.43739 ***	−1.95195 ***	−2.23629 ***	−2.45789 ***
*Saci2-8*	**All cesdtodes examined (12)**	−1.41048 *	−1.45125 *	−1.64427 *	−1.39480 *	−1.35879 *	−1.56165 *
	*S. erinaceieuropaei* (10)	−1.21924 *	−1.14093 *	−1.31248 *	−1.23207 *	−1.12958 *	−1.30622 *

^a^ Only sequences with copy numbers greater than five were subjected to the neutrality test at the intragenomic level. ^b,^*, *p* > 0.10; **, 0.10 > *p* > 0.05; ***, *p* < 0.05; and ****, *p* < 0.01. ^c^ *S. erinaceieuropaei* (16) includes both the *Se_Saci2-6a* and *Se-Saci2-6b* elements, whereas *S. erinaceieuropaei-b* (13) includes only the *Se-Saci2-6b* element of *S. erinaceieuropaei.*

**Table 3:** Mean p-distances within and between paralogous copies of cestode *Saci2*-like retrotransposons.

Element	Paralogous Group ^a^	Int Region				RT Region			
		*Se_Saci2* ^b^	*Sp_Saci2*	*Ss_Saci2*	*Li_Saci2*	*Se_Saci2*	*Sp_Saci2*	*Ss_Saci2*	*Li_Saci2*
***Saci2-1*** 0.396 ± 0.032 ^c.1^ 0.348 ± 0.016 ^c.2^	*Se_Saci2*	**0.073 ± 0.003**	0.121 ± 0.006	0.390 ± 0.032	0.572 ± 0.059	**0.069 ± 0.004**	0.111 ± 0.005	0.357 ± 0.018	0.489 ± 0.030
	*Sp_Saci2*		**0.140 ± 0.006**	0.390 ± 0.032	0.593 ± 0.062		**0.130 ± 0.008**	0.363 ± 0.018	0.509 ± 0.029
	*Ss_Saci2*			**0.585 ± 0.045**	0.728 ± 0.081			**0.547 ± 0.033**	0.643 ± 0.033
	*Li_Saci2*				**0.180 ± 0.008**				**0.174 ± 0.007**
***Saci2-2*** 0.379 ± 0.020 0.338 ± 0.017	*Se_Saci2*	**0.090 ± 0.004**	0.182 ± 0.009	0.565 ± 0.030	0.688 ± 0.039	**0.084 ± 0.005**	0.182 ± 0.013	0.492 ± 0.030	0.629 ± 0.042
	*Sp_Saci2*		**NC ^d^**	0.597 ± 0.033	0.729 ± 0.044		**NC**	0.543 ± 0.037	0.700 ± 0.049
	*Ss_Saci2*			**0.186 ± 0.007**	0.741 ± 0.043			**0.172 ± 0.008**	0.653 ± 0.041
	*Li_Saci2*				**0.544 ± 0.034**				**0.476 ± 0.039**
***Saci2-3*** 0.173 ± 0.006 0.164 ± 0.008	*Se_Saci2*	**0.122 ± 0.004**	0.113 ± 0.004	0.651 ± 0.029		**0.116 ± 0.006**	0.108 ± 0.006	0.618 ± 0.043	
	*Sp_Saci2*		**0.114 ± 0.008**	0.633 ± 0.030			**0.109 ± 0.011**	0.603 ± 0.044	
	*Ss_Saci2*			**NC**				**NC**	
***Saci2-4*** 0.202 ± 0.006 0.192 ± 0.010	*Se_Saci2*	**0.085 ± 0.003**	0.076 ± 0.004	0.552 ± 0.022		**0.086 ± 0.005**	0.070 ± 0.006	0.510 ± 0.031	
	*Sp_Saci2*		**NC**	0.523 ± 0.022			**NC**	0.457 ± 0.030	
	*Ss_Saci2*			**0.446 ± 0.025**				**0.429 ± 0.038**	
***Saci2-5*** 0.415 ± 0.017 0.383 ± 0.024	*Se_Saci2*	**0.093 ± 0.004**	0.117 ± 0.004		0.689 ± 0.030	**0.087 ± 0.006**	0.108 ± 0.006		0.631 ± 0.046
	*Sp_Saci2*		**0.129 ± 0.005**		0.722 ± 0.031		**0.118 ± 0.007**		0.677 ± 0.049
	*Li_Saci2*				**0.172 ± 0.007**				**0.161 ± 0.010**
***Saci2-6*** 0.425 ± 0.015 0.337 ± 0.019	*Se_Saci2*	**0.090 ± 0.003**	0.133 ± 0.004	0.721 ± 0.031	0.840 ± 0.037	**0.080 ± 0.005**	0.120 ± 0.007	0.621 ± 0.048	0.643 ± 0.046
	*Sp_Saci2*		**0.148 ± 0.006**	0.735 ± 0.031	0.879 ± 0.038		**0.136 ± 0.010**	0.635 ± 0.048	0.674 ± 0.047
	*Ss_Saci2*			**NC**	0.932 ± 0.044			**NC**	0.635 ± 0.048
	*Li_Saci2*				**0.031 ± 0.002**				**0.033 ± 0.003**
***Saci2-7*** 0.382 ± 0.011 0.370 ± 0.016	*Se_Saci2*	**0.026 ± 0.002**	0.083 ± 0.006	0.474 ± 0.017	0.412 ± 0.019	**0.027 ± 0.003**	0.101 ± 0.010	0.479 ± 0.026	0.402 ± 0.028
	*Sp_Saci2*		**NC**	0.507 ± 0.019	0.443 ± 0.021		**NC**	0.489 ± 0.027	0.418 ± 0.029
	*Ss_Saci2*			**0.361 ± 0.010**	0.542 ± 0.019			**0.359± 0.015**	0.508 ± 0.027
	*Li_Saci2*				**0.065 ± 0.003**				**0.068 ± 0.004**
***Saci2-8*** 0.137 ± 0.005 0.136 ± 0.007	*Se_Saci2*	**0.123 ± 0.004**	0.167 ± 0.006			**0.120 ± 0.007**	0.170 ± 0.010		
	*Sp_Saci2*		**0.170 ± 0.010**				**0.180 ± 0.015**		

^a^ The identity of each paralogous group is dustinguished by an abbreviated name of its donor species, as follows: Li, *Ligula intestinalis*; Se, *Spirometra erinaceieuropaei*; Sp, *Sparganum proliferum*; and Ss, *Schistocephalus solidus*. ^b^ Numerals categoring the cestode *Saci2* elements into families were omitted in the column titles for clarity. ^c^ Mean p-distances among all copies of each *Saci2* family calculated from from Int (c.1) and RT (c.2) regions. Standard deviations were estimated with 1000 bootstrap replicates. ^d^ NC: not calculated for paralogous groups represented by a single copy.

## Data Availability

The data used to support the findings of this study are available from the corresponding author upon request.

## Supplementary Materials

The following supporting information can be downloaded at: https://www.mdpi.com/article/10.3390/ijms26189061/s1.

## Abbreviations

The following abbreviations are used in this manuscript:

LTR	Long terminal repeat
TE	Transposable element
CDS	Coding DNA sequence
PBS	Primer binding site
TSD	Target site duplication
ORF	Open reading frame
Pol	Polyprotein
RT	Reverse transcriptase
Ts	Transition
Tv	Transversion
