# Supplementary material for: Structural and Evolutionary Analysis of Saci2-Like LTR Retrotransposons in Diphyllobothriidean Tapeworms
Source: Int J Mol Sci. 2025 Sep 17;26(18):9061. doi: 10.3390/ijms26189061 (PMC12471100; doi:10.3390/ijms26189061)
Supplement: Supplementary file 1 [file ijms-26-09061-s001.zip › Sparganum Rn_Suppl Figs-IJSM.pptx]

## Slide 1
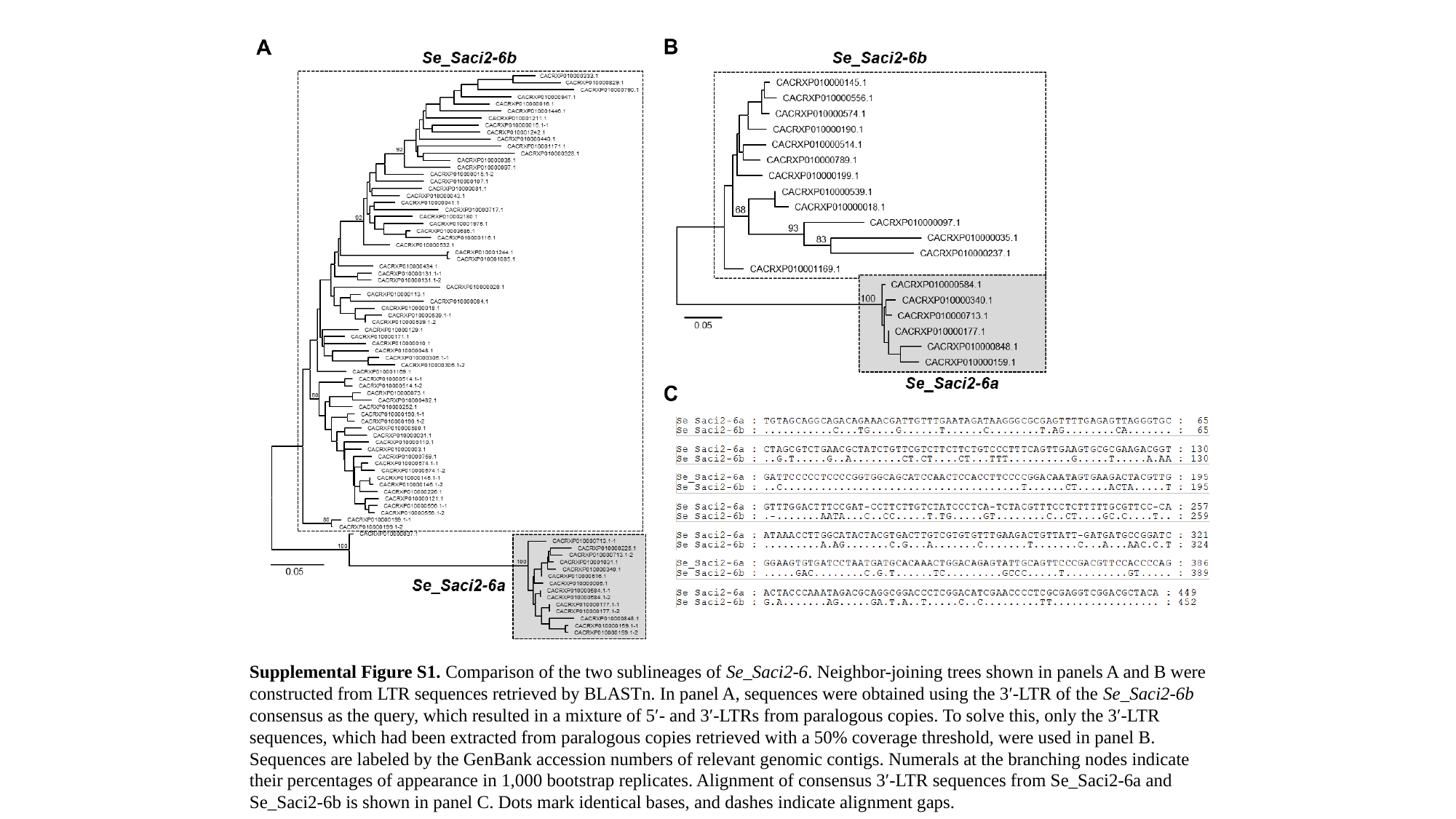

Supplemental Figure S1. Comparison of the two sublineages of Se_Saci2-6. Neighbor-joining trees shown in panels A and B were constructed from LTR sequences retrieved by BLASTn. In panel A, sequences were obtained using the 3′-LTR of the Se_Saci2-6b consensus as the query, which resulted in a mixture of 5′- and 3′-LTRs from paralogous copies. To solve this, only the 3′-LTR sequences, which had been extracted from paralogous copies retrieved with a 50% coverage threshold, were used in panel B. Sequences are labeled by the GenBank accession numbers of relevant genomic contigs. Numerals at the branching nodes indicate their percentages of appearance in 1,000 bootstrap replicates. Alignment of consensus 3′-LTR sequences from Se_Saci2-6a and Se_Saci2-6b is shown in panel C. Dots mark identical bases, and dashes indicate alignment gaps.

## Slide 2
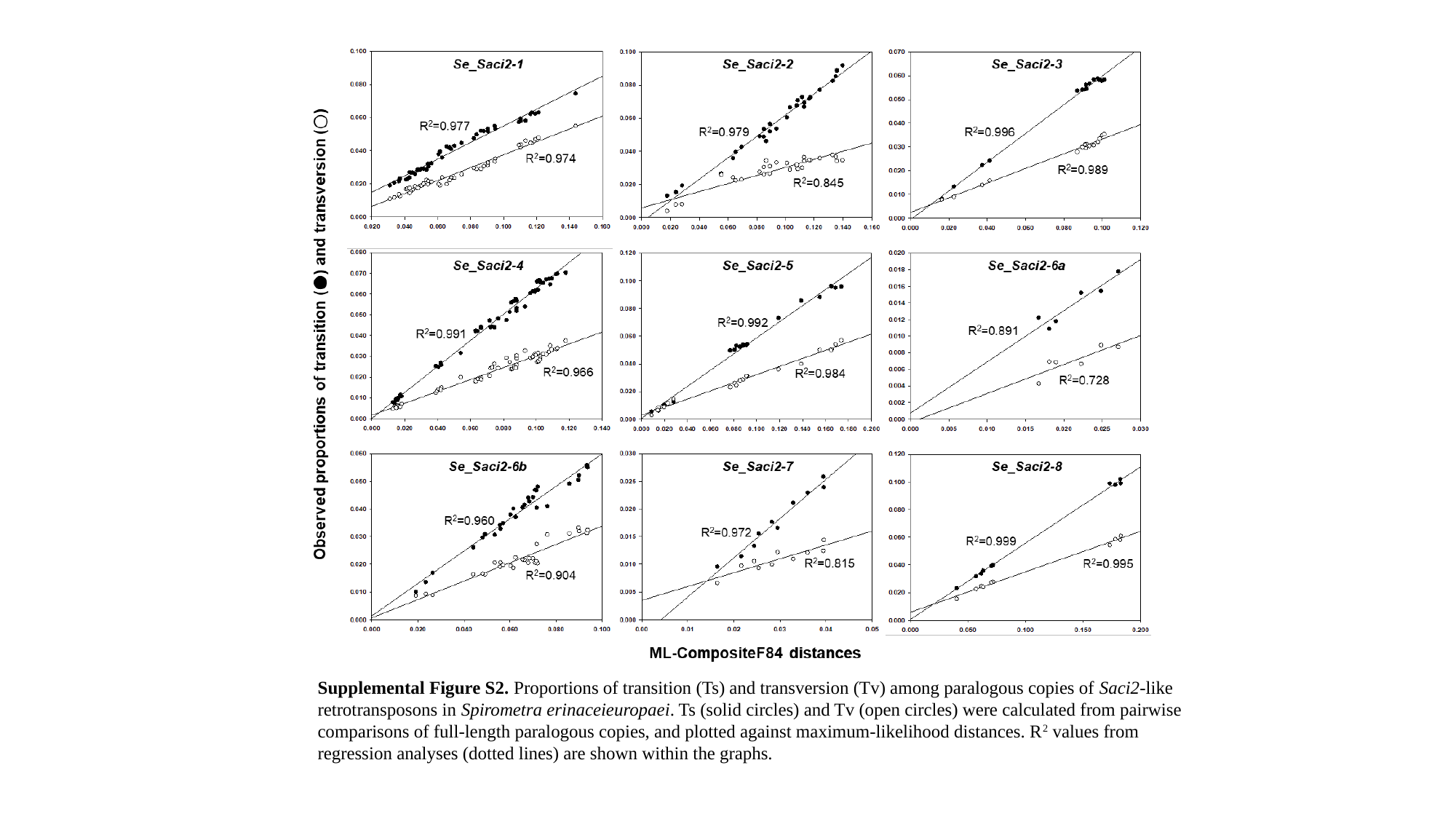

Supplemental Figure S2. Proportions of transition (Ts) and transversion (Tv) among paralogous copies of Saci2-like retrotransposons in Spirometra erinaceieuropaei. Ts (solid circles) and Tv (open circles) were calculated from pairwise comparisons of full-length paralogous copies, and plotted against maximum-likelihood distances. R2 values from regression analyses (dotted lines) are shown within the graphs.

## Slide 3
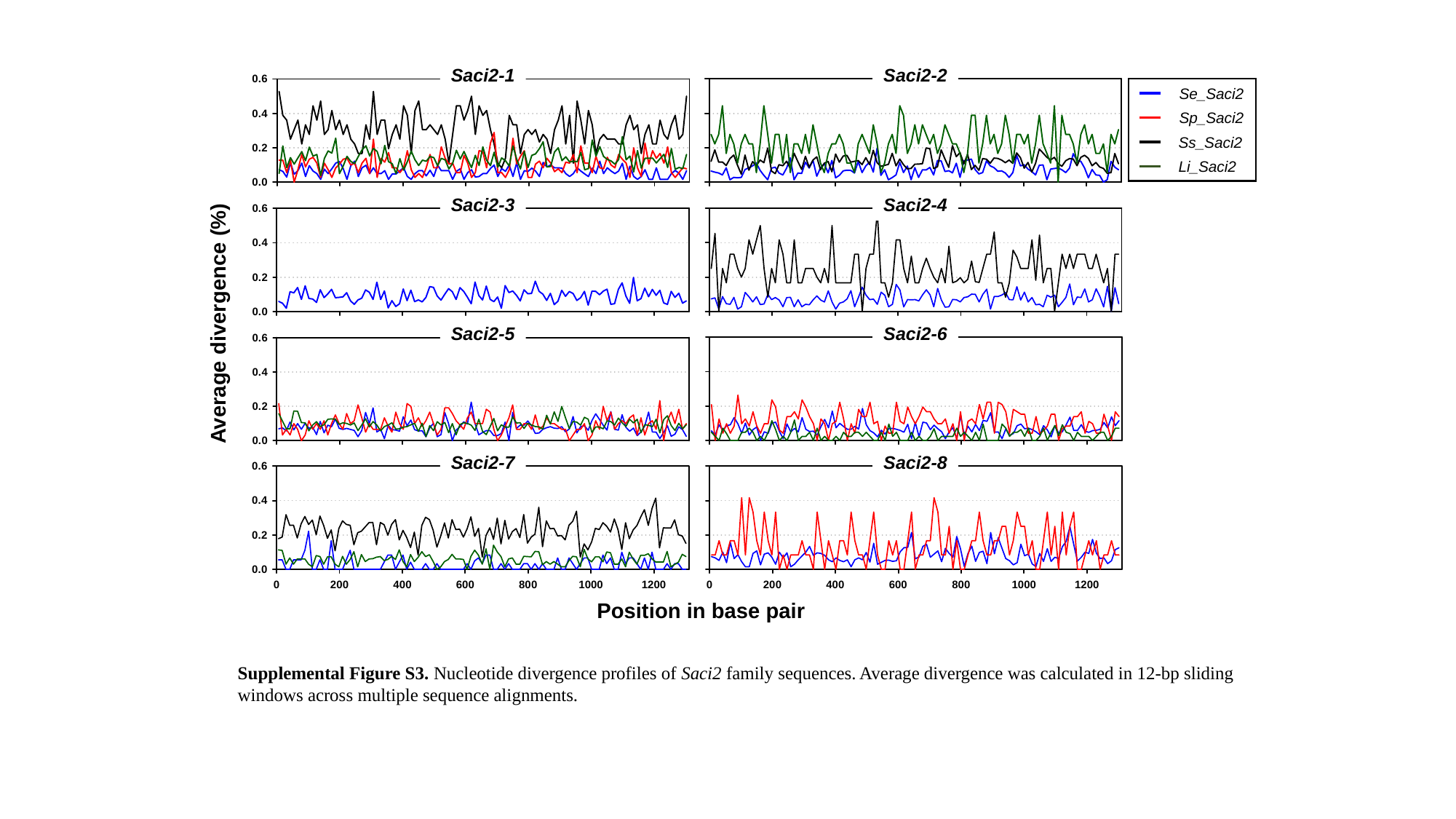

Saci2-1
Saci2-2
Se_Saci2
Sp_Saci2
Ss_Saci2
Li_Saci2
Saci2-3
Saci2-4
Average divergence (%)
Saci2-5
Saci2-6
Saci2-7
Saci2-8
Position in base pair
Supplemental Figure S3. Nucleotide divergence profiles of Saci2 family sequences. Average divergence was calculated in 12-bp sliding windows across multiple sequence alignments.

## Slide 4
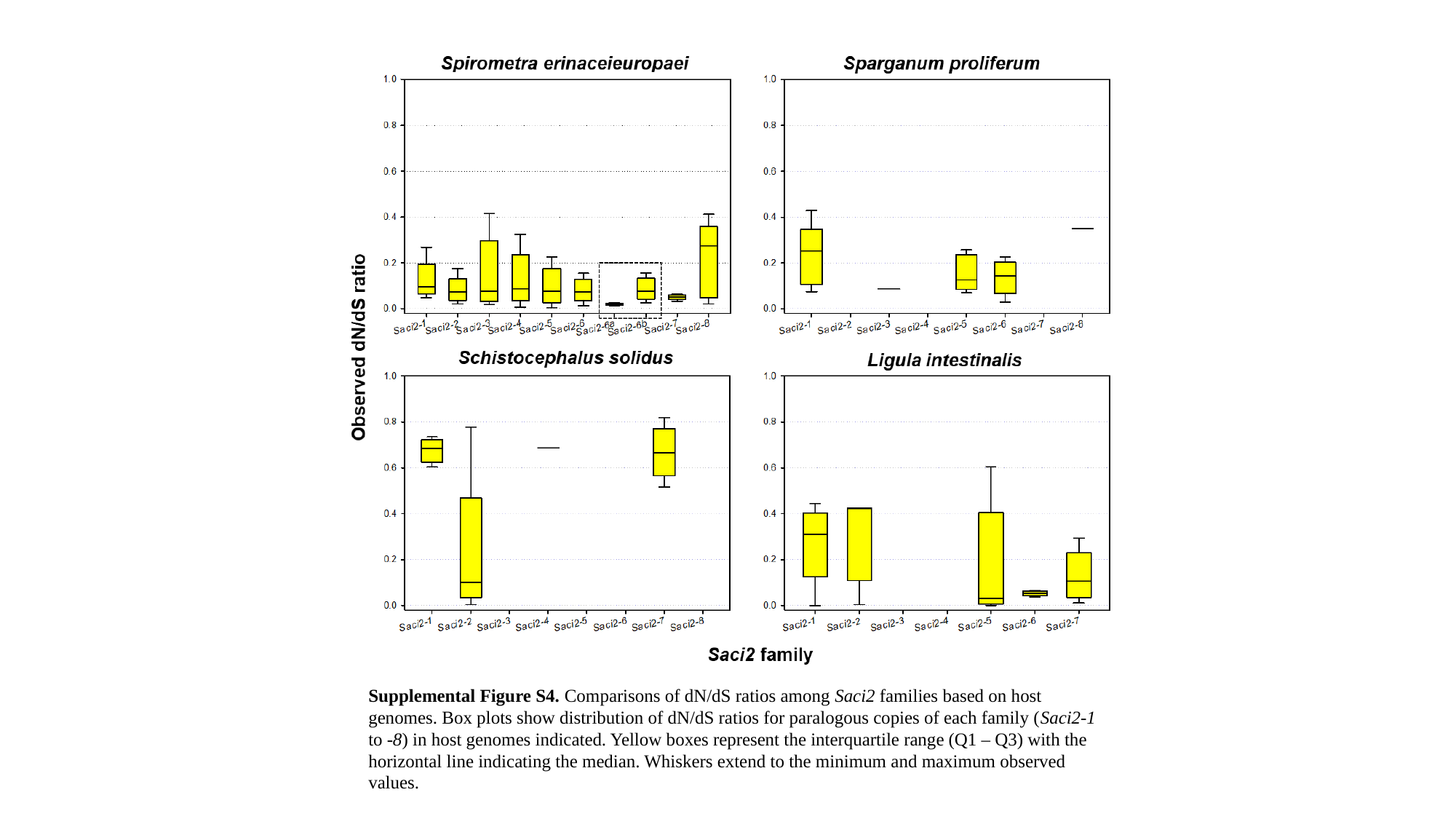

Supplemental Figure S4. Comparisons of dN/dS ratios among Saci2 families based on host genomes. Box plots show distribution of dN/dS ratios for paralogous copies of each family (Saci2-1 to -8) in host genomes indicated. Yellow boxes represent the interquartile range (Q1 – Q3) with the horizontal line indicating the median. Whiskers extend to the minimum and maximum observed values.

## Slide 5
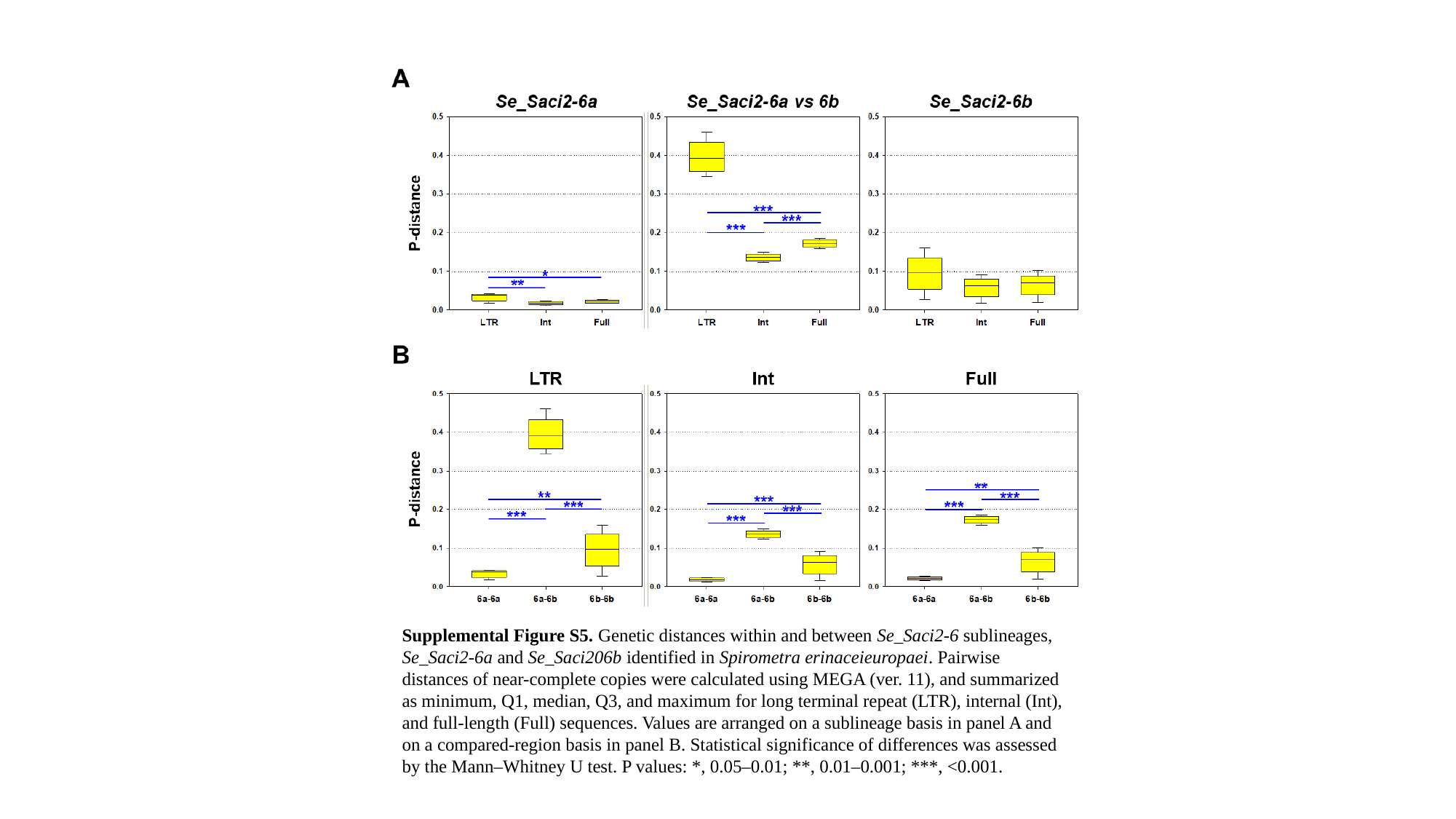

Supplemental Figure S5. Genetic distances within and between Se_Saci2-6 sublineages, Se_Saci2-6a and Se_Saci206b identified in Spirometra erinaceieuropaei. Pairwise distances of near-complete copies were calculated using MEGA (ver. 11), and summarized as minimum, Q1, median, Q3, and maximum for long terminal repeat (LTR), internal (Int), and full-length (Full) sequences. Values are arranged on a sublineage basis in panel A and on a compared-region basis in panel B. Statistical significance of differences was assessed by the Mann–Whitney U test. P values: *, 0.05–0.01; **, 0.01–0.001; ***, <0.001.

## Slide 6
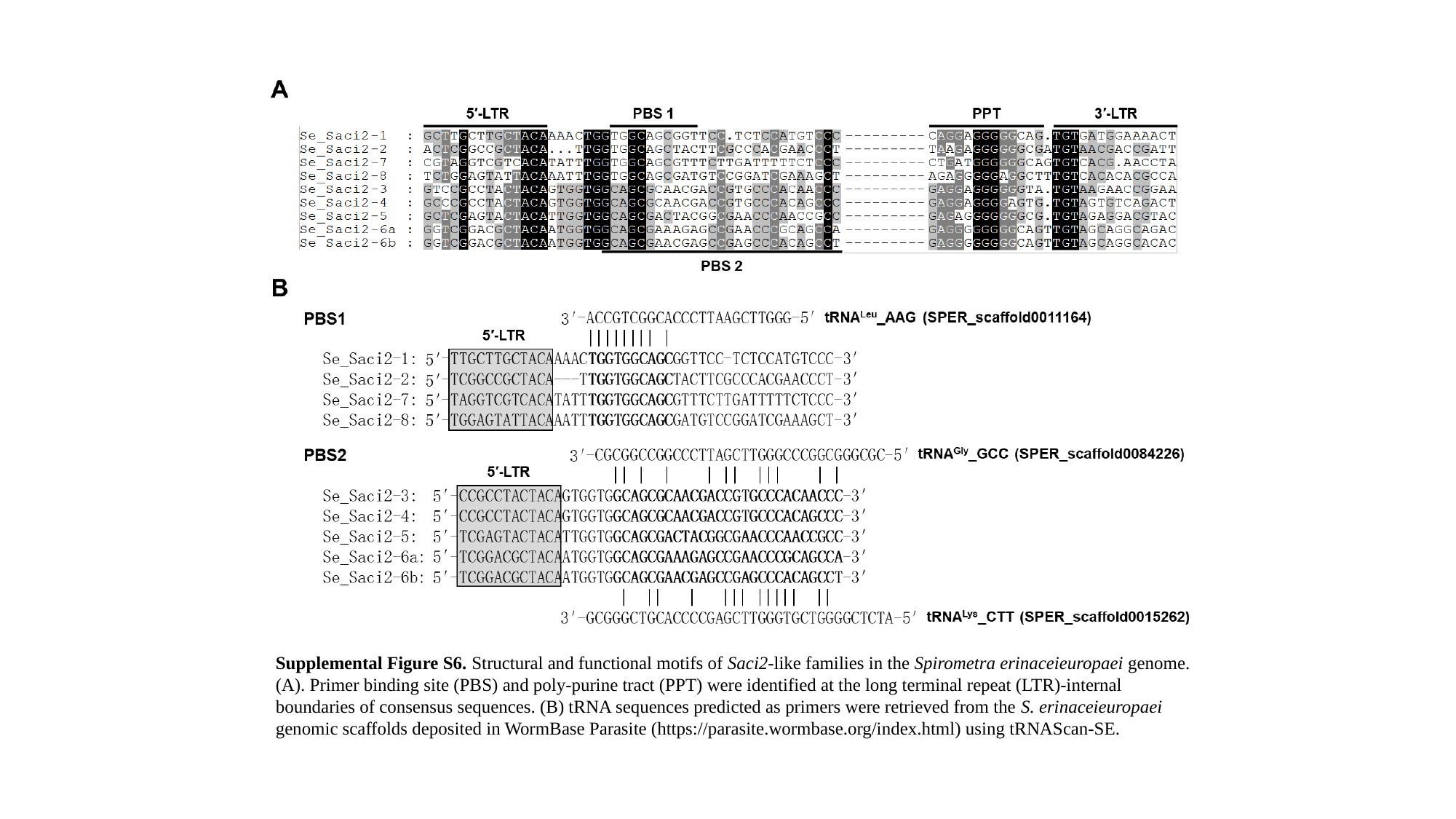

Supplemental Figure S6. Structural and functional motifs of Saci2-like families in the Spirometra erinaceieuropaei genome. (A). Primer binding site (PBS) and poly-purine tract (PPT) were identified at the long terminal repeat (LTR)-internal boundaries of consensus sequences. (B) tRNA sequences predicted as primers were retrieved from the S. erinaceieuropaei genomic scaffolds deposited in WormBase Parasite (https://parasite.wormbase.org/index.html) using tRNAScan-SE.
